# Design of a 3D nanowire-CuO/LDH@FeNi-γAl_2_O_3_ catalyst and its synergistic mechanism for accelerated dye degradation in wastewater

**DOI:** 10.1039/d5ra06641g

**Published:** 2026-01-19

**Authors:** Guene L. Razack, Jie Ding, Xian Zhao, Ya-Ni Zang, Chen-Hao Cui, Wen-Shuo Wang, Ji-Wei Pang, Lu-Yan Zhang, Worou Chabi Noel, Assogba Dou Rached, Nan-Qi Ren, Shan-Shan Yang

**Affiliations:** a National Engineering Research Center for Safe Disposal and Resources Recovery of Sludge, Harbin Institute of Technology Harbin 150090 China shanshanyang@hit.edu.cn dingjie123@hit.edu.cn; b State Key Laboratory of Urban-rural Water Resource and Environment, School of Environment, Harbin Institute of Technology Harbin 150090 China; c Laboratory of Materials and Structures (LAMS). Graduate School of Civil Engineering Véréchaguine 02 BP 244 Cotonou Benin; d Laboratory of Water Science and Technology, National Water Institute (INE), University of Abomey-Calavi 01 BP 526 Cotonou Benin; e Harbin Corner Science & Technology Inc. Harbin 150023 China; f School of Environmental Science and Engineering, Yancheng Institute of Technology Yancheng 224051 China

## Abstract

Three-dimensional (3D) particle electrode oxidation technology is an effective advanced oxidation technology for degrading organic pollutants. In this study, a series of γAl_2_O_3_-supported FeNiCu-layered double hydroxide/CuO composite catalysts (γAl_3_O_3_ × FNCL@CuO) were synthesized, with the aim of optimizing the electrode preparation process and enhancing the electrocatalytic degradation efficiency for organic dye wastewater. Although γAl_2_O_3_ itself exhibits relatively low electronic activity, it serves as an effective catalytic support with notable performance-promoting properties, thus representing a valuable subject for investigation. The materials were characterized using X-ray diffraction, scanning electron microscopy, and Fourier-transform infrared spectroscopy. The composite electrode γAl_3_O_3_ × FNCL@CuO was compared with a pure γAl_2_O_3_ 3D particle electrode in terms of performance. The results demonstrated that the composite electrode significantly outperformed the pure γAl_2_O_3_ electrode in pollutant removal and overall electrocatalytic activity. The degradation rates for rhodamine B (RhB), acid yellow (AY), and methylene blue (MB) reached 98.82%, 99.99%, and 98.97%, respectively. Furthermore, comparisons between two-dimensional (2D) and 3D systems revealed that the porous 3D structure facilitates the oxidation process by increasing the contact interface between active sites and reactant molecules. Quenching experiments confirmed that radical scavengers had only a minor inhibitory effect on RhB degradation in the 3D system. In conclusion, γAl_3_O_3_ × FNCL@CuO shows promising potential for application in the electrochemical treatment of organic wastewater and merits further research.

## Abstract 2

The heterojunction of γAl_2_O_3_ × FNCL@CuO particles was elucidated. The excellent degradation of dyes and stability of particles were demonstrated. The mechanism of dye removal in the 3D system has been elucidated.

## Graphical abstract

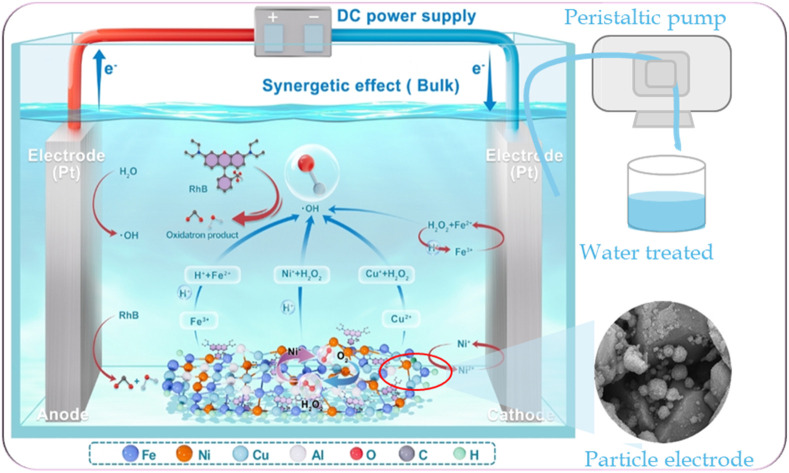

## 1. Introduction

Dye effluent, a hazardous form of industrial wastewater, has gathered significant research focus due to its unique characteristics and potential environmental dangers.^1^ Each year, approximately 175000 tons of dyes are released indiscriminately into the global water supply.^2,3^ According to the United Nations World Water Development Report, more than 80% of wastewater from various industries is discharged directly into the environment without sufficient treatment.^1^ The release of textile waste into natural water bodies or through land composting contaminates vital resources with harmful substances, including chemicals, polymers, dyes, and other carcinogenic materials.^4,5^ In response to this crisis, industrialized countries have initiated scientific efforts to address the escalating pollution from wastewater. The increasing demands from major industrial enterprises, such as the textile industry, drive these efforts.^4^ Given the scarcity of water resources, developing strategies that implement diverse remediation strategies is essential to improving sustainability and wastewater treatment.^6^

Indeed, wastewater treatment involves various techniques, including 3D electrochemistry, flocculation, coagulation, adsorption, biological treatment, and photocatalytic oxidation.^7^ Electrocatalytic (EP) processes, which involve redox and adsorption, offer a promising solution for decentralized systems due to their high *in situ* efficiency, capacity to generate diverse oxidants, compact size, and ease of operation and maintenance.^8^ The efficient oxidation of refractory organic pollutants can be achieved through rational and optimized reactor and electrode design, a highly promising technology for efficiently treating industrial wastewater. However, in conventional two-dimensional electrocatalysis, the transportation of electrons is the sole phenomenon observed across the electrode plate. This results in low electrocatalytic oxidation efficiency, high energy consumption, and a large apparatus footprint due to limited current efficiency and the inherently narrow contact area of the electrode plate.^9^ Therefore, in wastewater treatment, it is essential to carefully regulate the composition, morphology, and size of both cathode and anode materials.^8^

The 3D electrocatalytic oxidation method is the most effective and widely used method for removing organic pollutants that are otherwise difficult to eliminate.^7,10^ The mechanisms by which 3D electrode reactors remove contaminants include adsorption, electro-adsorption, electro-flocculation, redox reactions, and the catalytic production of effective oxidants.^11^ In recent years, significant progress in the field of material science has presented a remarkable opportunity to address the prevailing challenges in wastewater treatment. For instance, a 3D free-standing integrated electrode was constructed using multiple virtually single-crystal TiO_2−*δ*_N_*δ*_ nanowire arrays.^12^ According to Zhang *et al.*,^13^ the oxidation of *p*-aminophenol wastewater was achieved using a Ti–Si–Sn–Sb/GAC material in a 3D electro-oxidation reactor.^14^ In the simultaneous catalytic degradation of mercury and chlorobenzene, CuO-modified CeO_2_–Al_2_O_3_ catalysts with a 3D porous structure demonstrated remarkable activity and stability.^15^ In the context of total water splitting within an alkaline environment, layered double hydroxides (LDHs) composed of earth–abundant transition metals exhibit considerable potential as electrocatalysts. This is attributed to their high intrinsic activity, abundance, and cost-effectiveness.^16^ However, the attachment of the powder catalyst to the conductive substrate typically requires the use of a polymer binder, which can lead to the aggregation of LDH nanosheets and increased contact ohmic resistance between the powder and the substrate.^16^ Several effective strategies have been identified, including the direct bonding of the catalyst to the conductive framework to minimize contact ohmic resistance, the reduction of the catalyst size to increase the surface area and expose more active sites, and the creation of a multi-channel, 3D hierarchical structure to facilitate mass transfer.^16^ Additionally, the porous nature of these catalyst structures offers several advantageous properties. These include enhanced electrical conductivity, substantial mechanical and chemical stability, and an extensive surface area, which in turn increases active sites and improves reactant accessibility.^17,18^ Then, to enhance the electrochemical functionality of electrodes and the product morphology, it is imperative to construct a rational three-dimensional CuO nanowire on chloroform (CF) with robust adhesion. This will facilitate the development of novel nanostructure particle electrodes 3D to treat organic wastewater.

The fabrication of 3D porous foam-type nanostructures has gained attention as an effective strategy that significantly enhances electrocatalytic activity and accelerates the kinetics of water splitting, owing to their larger electroactive surface area, which offers more active sites and facilitates catalyst loading.^19^ For instance, nickel foam (Nif) serves a dual purpose as both a matrix and a current collector when used as a substrate, enabling the growth of active electrocatalysts on its surface. Conversely, copper foam (Cuf) has been demonstrated to be more cost-effective than nickel foam and exhibits superior conductivity and ductility. In the process of water electrolysis, copper foam is frequently utilized as a substrate for the development of active materials.^19–21^ By avoiding high-temperature pyrolysis, these techniques provide a framework for fabricating LDH-based electrocatalysts that retain intrinsic characteristics derived from MOFs.^22^ Metal LDH can also be combined with γAl_2_O_3_ due to the latter's status as a highly versatile substrate material, which can be attributed to its notable physical and chemical properties, including porosity, an excellent specific surface area, and exceptional thermal stability. It has been determined that the substance in question possesses a more substantial prospective role in catalysis as a catalyst support. It is especially well-suited for this purpose,^23^ wastewater treatment. Furthermore, the huge specific surface area of Co–Ce–Zr/γ-Al_2_O_3_ offers additional active sites. As such, a considerable amount of ˙OH can be created in the reaction, which significantly improves the degradation rate of CIP. Based on the parameters above, it is hypothesized that Co–Ce–Zr/γ-Al_2_O_3_ can demonstrate exceptional catalytic performance.^24^ However, γAl_3_O_3_ × FNCL@CuO has not yet been applied in the 3D electrocatalysis of organic wastewater treatment.

This study presents a systematic investigation into the degradation of organic dye micropollutants in wastewater using a 3D electrocatalytic system activated by a γAl_2_O_3_-supported catalyst composed of nanowire CuO and a tri-metallic FeNiCu-layered double hydroxide (LDH). The kinetics of pollutant degradation by the γAl_3_O_3_ × FNCL@CuO 3D particle electrode were examined under varying experimental conditions and in the presence of different water constituents. Furthermore, the primary reaction pathways were elucidated, with emphasis on the generation of reactive species and the enhancement of electron transfer, which collectively contribute to the efficient removal of model pollutants-methylene blue (MB), acid yellow (AY), and rhodamine B (RhB). A mechanism for periodate activation by γAl_3_O_3_ × FNCL@CuO under 3D conditions is proposed based on theoretical calculations, along with a plausible degradation pathway for the target dyes. In light of growing environmental concerns, these findings provide new insights into the activation and optimization of advanced oxidation processes using 3D particle electrode systems.

## 2. Materials and methods

### 2.1. Material

The chemicals, reagents utilized, and additional details can be found in the SI in Test S1.

### 2.2. Synthesis of the 3D electrocatalyst

The 3D electrocatalyst, γAl_2_O_3_ × FNCL@CuO, consisting of transition and post-transition metals, was synthesized *via* the preparation of two solutions, denoted as A (FeNiCu-LDH@CuO) and B(γAl_2_O_3_ × FNCL@CuO). Initially, Cu(OH)_2_ (1 × 3 cm^2^) was synthesized. Its surface was pretreated with ethanol, acetone, and hydrochloric acid, followed by rinsing with demineralized water. The Cu(OH)_2_ was then immersed in a 40 mL solution (2.0 M NaOH and 0.1 M (NH_4_)_2_S_2_O_8_) for 25 min at 25 °C. Afterward, the material was rinsed with ethanol, DI water, and dried at 85 °C for 8 h, yielding Cu(OH)_2_. Afterward, it was heated to 280 °C for 3 hours to form nanowire-CuO.

Then, the synthesis of FeNiCu-LDH@CuO was performed using a solvothermal process with a Fe/Ni molar ratio of 1 : 1. The concentrations of Fe(NO_3_)_2_·6H_2_O and Ni(NO_3_)_2_·6H_2_O were determined to be 0.1621 mmol and 0.3742 mmol, respectively, in a 70 mL solution composed of 15 mL of *N*,*N*-dimethylformamide and 50 mL of ethanol. The solution was stirred for 1 h after the addition of 5 mL of deionized water (DI) to prepare solution A. The mixture was then placed in an autoclave and heated at 130 °C for 11 hours. Then, the resultant FeNiCu-LDH@CuO material was washed multiple times with ethanol and DI water under vacuum, dried at 85 °C for 12 hours, and designated as FNCL@CuO, and all chemical designations were listed in Table S1.

Separately, γAl_2_O_3_ was washed with ethanol and DI water using ultrasonic vibration for 1 hour, followed by drying at 95 °C for 7 hours to yield pretreated γAl_2_O_3_. Various masses (5, 8, 15, 20, and 30 g) of pretreated γAl_2_O_3_ were then added to solution A to obtain solution B. Solution B was synthesized using the same solvothermal method as solution A, resulting in γAl_2_O_3_ × FNCL@CuO. This material was calcinated at 300, 400, 500, and 600 °C temperatures.

### 2.3. Characterizations

A scanning electron microscope (SEM, Ultima IV, Rigaku, Japan) and a Transmission electron microscope (TEM, Ultima IV, Rigaku, Japan) were used to examine the surface and internal morphology of both fresh and used electrocatalysts. FT-IR spectroscopy was conducted at room temperature using a Rigaku Ultima IV spectrometer (Japan) to analyze the functional groups in the materials, spanning the range of 400 cm^−1^ to 4000 cm^−1^. The crystal properties were investigated using XRD (Ultima IV, Rigaku, Japan) with Cu Kα irradiation, which facilitated the investigation of valence state changes in surface elements. The elemental composition was further analyzed using XPS (Ultima IV, Rigaku, Japan). The pore architectures of the materials were analyzed through EDS and SEM (Ultima IV, Rigaku, Japan). The BET surface area and pore properties were determined using N_2_ adsorption–desorption isotherms on a BJH-Builder Ultima IV instrument from Rigaku, Japan. Electrocatalytic oxidation measurement of the materials was used to analyze their performance.

The pH value was adjusted using 0.1 M HCl or NaOH and detected with a DDS-307A pH meter (Electrical Scientific Instruments Ultima IV, Rigaku, Japan). The pH of the reactor was determined when the steel electrode plates, along with the Pt anode and cathode, were submerged to a depth of three centimeters in 250 mL of dye wastewater solution containing RhB, MB, and AY. The electrolytic cell power was connected to a specified mass of the particle electrode. The removal efficiency was measured at intervals ranging from 0 to 40 min.

UV-Vis spectrophotometers were employed to determine the absorption or transmission of light through a medium to assess the removal efficiency using the following formula (Test S2). The 3D electrochemical reactor was a clear rectangular tank made of biological glass, measuring 40 cm × 20 cm × 30 cm. A specified number of particle electrodes was inserted into the anode and cathode electrodes inside the reactor. The anode and cathode plates were arranged parallel and perpendicular to one another, maintaining 5 cm. Dimensionally stable Pt electrodes measuring 25 cm × 15 cm × 0.2 cm were used as the cathode and anode. The degradation rates of RhB, MB, and AY (denoted as *X*_RhB_, *X*_MB_, and *X*_AY_) were calculated using eqn (S1–S7). Detailed procedures for electrocatalyst sample preparation and electroanalytical measurements are provided in SI, Test S3.

## 3. Results and discussion

### 3.1. Synthetization of γAl_2_O_3_ × FNCL@CuO

Fig. S2 demonstrates the adsorption performance of organic pollutant dyes (RhB, MB, and AY) by γAl_2_O_3_ and with a subtract particle electrode (3D). The adsorption time was 40 min, and the removal efficiencies of γAl_2_O_3_ for RhB, MB, and AY were 8.08, 8.95, and 7.78%, respectively. Without using γAl_2_O_3,_ the removal capacities increased to 10.53, 12.82, and 10.62% respectively. However, when using the γAl_2_O_3_ 3D particle electrode, the removal capacities for RhB, MB, and AY increased further to 15.6, 19.03, and 16.3%, respectively. Fig. S3 presents the catalytic performance of FNCL_0.1621_@CuO, FNCL_0.2733_@CuO, and FNCL_0.3742_@CuO using the 3D electrode to remove RhB, MB, and AY from water. The removal efficiencies were 70.6, 84.27, and 76.8% respectively. The particle electrode FNCL_0.2733_@CuO exhibited better degradation performance than FNCL_0.1621_@CuO and FNCL_0.3742_@CuO.

Subsequently, the various catalysts FNCL_0.2733_@CuO, γAl_2_O_3_ × FNCL_5_@CuO, γAl_2_O_3_ × FNCL_8_@CuO, γAl_2_O_3_ × FNCL_15_@CuO, γAl_2_O_3_ × FNCL_20_@CuO, and γAl_2_O_3_ × FNCL_30_@CuO was enhanced by incorporating γAl_2_O_3_. These varied particle electrodes were tested with the 3D electrodes to remove RhB from water. Thus, the removal performance shown in Fig. S4 was 55.55, 63.7, 85.85, 90.6, and 50.96%, respectively. Among these, the γAl_2_O_3_ × FNCL_20_@CuO electrode exhibited the highest reduction of RhB. The γAl_2_O_3_ × FNCL_20_@CuO particle electrode was heated at (300, 400, 500, and 600 °C) and labeled as γAl_2_O_3_ × FNCL_20_@CuO-300, γAl_2_O_3_ × FNCL_20_@CuO-400, γAl_2_O_3_ × FNCL_20_@CuO-500, and γAl_2_O_3_ × FNCL_20_@CuO-600. The removal performance of RhB from wastewater was investigated using these calcined electrodes, as illustrated in Fig. S5. The removal performance using the 3D electrode technology was 79.52, 96.69, 88.38, and 85.30%, respectively. The high removal of RhB was achieved with the γAl_2_O_3_ × FNCL_20_@CuO-400 particle electrode, following calcination at 400 °C. The performance removal of the catalysts in the 3D system was summarized in Table S2.

### 3.2. Structure and properties of γAl_2_O_3_ × FNCL@CuO

**Fig. 1:**
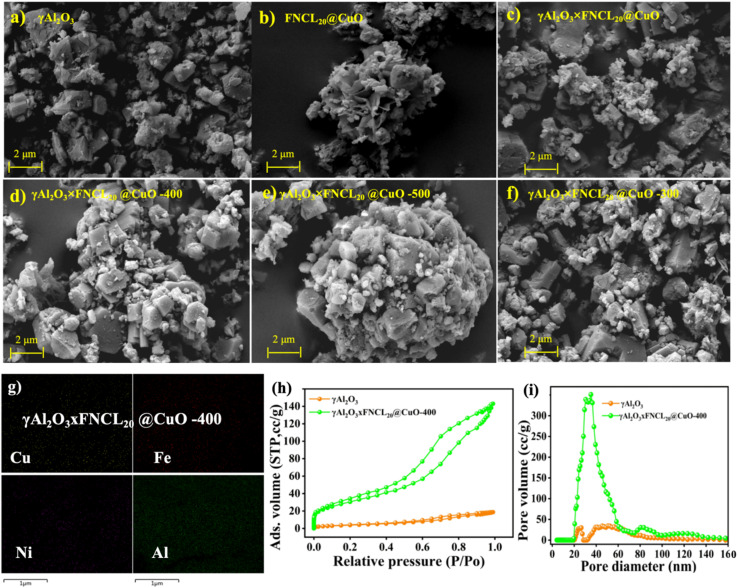
Scanning electron microscope (SEM) images at different modifications and different temperatures, at (a) γAl_2_O_3,_ (b) FNCL@CuO, (c) γAl_2_O_3_ × FNCL_20_@CuO, (d) γAl_2_O_3_ × FNCL_20_@CuO-300, (e) γAl_2_O_3_ × FNCL_20_@CuO-400, (f) γAl_2_O_3_ × FNCL_20_@CuO-500, and (g) EDS-mapping of γAl_2_O_3_ × FNCL_20_@CuO-400, (h) relation pressure, and (i) pore diameter of γAl_2_O_3_ and γAl_2_O_3_ × FNCL_20_@CuO-400.

As illustrated in Fig. 1, the SEM image of γAl_2_O_3_ × FNCL@CuO was measured at a resolution of 2 µm. The surface of γAl_2_O_3_ exhibits a rough and uneven appearance, characterized by the presence of numerous irregularly raised structures reminiscent of burrs.^9^ The porous structure of γAl_2_O_3_ has been demonstrated to enhance mass transfer efficiency during the reaction and facilitate material loading.^24^ Thus, in Fig. 1a–c, numerous micro-spherical crystals and a few needle-like cubic crystal formations appear on the convex structure of γAl_2_O_3_ × FNCL@CuO following the loading of nanowire CuO and double-layer FeNi-LDH. As illustrated in Fig. S6, the structural modification of Al_2_O_3_ into nanometer-sized cubes is evident.^25^Fig. 1d and e demonstrates that more crystals form on the surface of γAl_2_O_3_ × FNCL@CuO compared to γAl_2_O_3_ and FNCL@CuO, which are catalytically active substances. Distinct needle-bed structures are observed on the surface of γAl_2_O_3_ × FNCL@CuO formed by nanowire-CuO double-layer hydrogen FeNi at calcination temperatures of 400 °C and 500 °C. A microsphere convex structure is also produced under high-temperature conditions, similar to the nanowire FNCL@CuO. Consequently, an increase in calcination temperature results in an increase in the number of microspheres observed on the surface of the nanometer-sized cubes of the electrode particles, as illustrated in Fig. S7.^25^

The elemental mapping analysis (Fig. 1g) confirms the successful loading of O, Al, Fe, Ni, and Cu onto γAl_2_O_3_, and the physical properties of the particle are summarized in Table S3. The comparative analysis of the physical properties of γAl_2_O_3_ × FNCL_20_@CuO-400 and γAl_2_O_3_ shows significant changes in the pore structure and surface area. The isotherms of both γAl_2_O_3_ and γAl_2_O_3_ × FNCL_20_@CuO-400 show type IV adsorption branches, as shown in Fig. 1h and i. The BET surface area for γAl_2_O_3_ and γAl_2_O_3_ × FNCL_20_@CuO-400 was 19 m^2^ g^−1^ and 144 m^2^ g^−1^, respectively. The increased surface area probably facilitates the interaction of the metal with the surface-active regions. In addition, the average pore size of γAl_2_O_3_ × FNCL_20_@CuO-400 increased from 20 nm to 40.3 nm by doping with Fe, Ni, and nanowire CuO (Table S4). This doping also led to substantial changes in pore volume, which exceeded that of γAl_2_O_3_ (Table S4). These results suggest that Fe, Ni, and nanowire-CuO doping significantly enhanced the specific surface area (*S*_BET_) of the original γAl_2_O_3_ catalyst.^26^

**Fig. 2:**
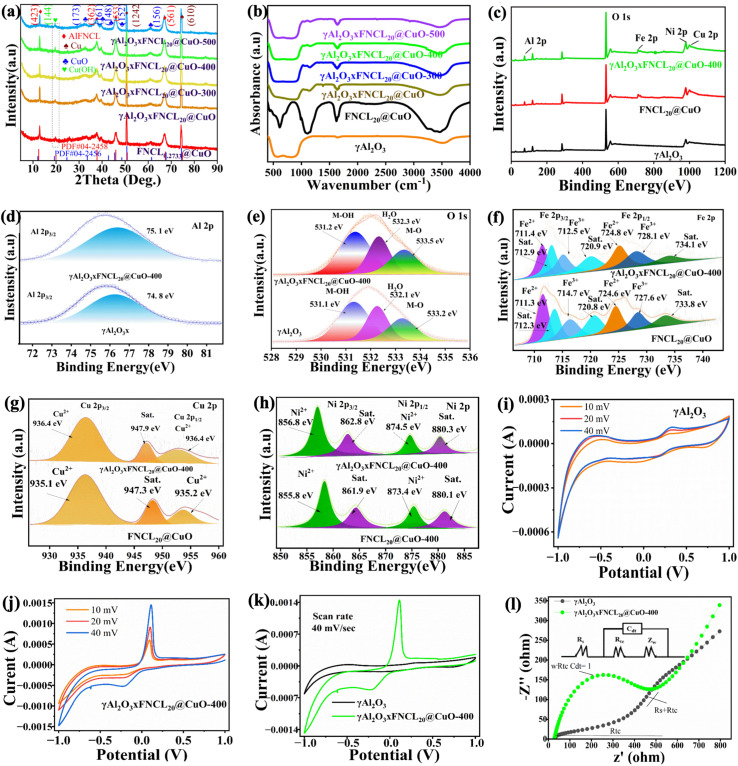
Characterization of the particle electrode of γAl_2_O_3_ and γAl_2_O_3_ × FNCL_20_@CuO-400. (a) XRD spectra; (b) FTIR spectra; (c) XPS spectra; (d) O 1s; (e) Al 2p; (f) Fe 2p, (g) Cu 2p; (h) Ni 2p; (i–k) CV curve, and (l) EIS curve.

The preliminary investigation into the crystallinity of the FNCL@CuO samples demonstrated that an enhancement was observed with increasing dosage of the raw material,^27^ as illustrated in Fig. S8. Thus, the particle γAl_2_O_3_ × FNCL_20_@CuO was studied through X-ray diffraction, with the results demonstrated in Fig. 2a. As the heating of the raw material increased, the γAl_2_O_3_ × FNCL_20_@CuO samples exhibited enhanced crystallinity. The diffraction peaks of FNCL@CuO at 12.88, 37.64, 48.29, 51.03, 61.52, 67.08, and 74.32° correspond to the crystal planes (423), (173), (148), (152), (1242), (156), and (610) respectively. Additionally, the diffraction peaks at 19.51, 37.6, 39.52, 45.91, and 67.03° (ref. 28) correspond to the γAl_2_O_3_ × FNCL_20_@CuO crystal planes (144), (362), (161), (553), and (558), respectively. This could be attributed to an incomplete reaction during the annealing process between Cu(OH)_2_ and CuO.^28^

As illustrated in Fig. 2b, the FTIR spectra of FNCL_20_@CuO and γAl_2_O_3_ × FNCL_20_@CuO were highly similar. The FTIR analysis determined the surface functional groups of γAl_2_O_3_ × FNCL_20_@CuO following modification at Al_2_O_3_ calcination temperatures of 300, 400, and 500 °C. The stretching vibrations of hydroxyl (˙OH) groups interacting with the surface metal ions were associated with the peaks at approximately 3405 cm^−1^ and 1627 cm^−1^.^23,29^ The γAl_2_O_3_ × FNCL_20_@CuO spectra exhibited several unique absorption peaks in comparison to the γAl_2_O_3_ spectrum. As posited by Sun *et al.*,^30^ the peaks at 3430 cm^−1^ and 1630 cm^−1^ were attributed to the straining and bending vibrations of HO–H,^31^ respectively, due to free and structured water. The racking vibration of Fe–O was identified as the cause of the band at 450 cm^−1^,^32^ while the O–Ni–O deformation vibration was linked to the peak at 610 cm^−1^. The straining vibration of Cu–O was responsible for the absorption peak at 670 cm^−1^, and the characteristic band of Cu–O–Cu was observed at 750 cm^−1^. The peak intensity increased with temperature. The prolonged vibration of the C–O bond was identified as the source of the absorption peak at 1070 cm^−1^.^33^ Furthermore, as noted by Zuo *et al.*,^34^ the bands in the 400–950 cm^−1^ range correspond to the straining vibrations of tetrahedral metal–oxygen bonds. The presence of Fe and Ni oxides in Al_2_O_3_ was indicated by the straining vibrations of F–O, Ni–O, and Cu–O–Cu and the deformation vibrations of O–Fe–O and O–Ni–O.^35^ After calcination, a significant increase in the peak intensity at 582 cm^−1^ was observed, which is probably due to the altered electronic structure of FNCL@CuO caused by the incorporation of Al^3+^. XPS analysis confirmed the elemental valence states of γAl_2_O_3_ × FNCL_20_@CuO synthesized under optimal conditions.

The survey spectrum (Fig. 2c) reveals the presence of Al, Cu, Fe, O, and Ni. The Al 2p peaks at 75.1 eV (Al 2p_3/2_) are evident in Fig. 2d. The high-resolution O 1s spectrum (Fig. 2e) shows three distinct peaks at 532.1, 533.2, and 531.1 eV, corresponding to adsorbed H_2_O on the sample surface,^36^ metal–oxygen bonding (M–O), and metal–hydrogen–oxygen bonding (M–OH), respectively.^37^ A new peak at 533.5 eV was observed in γAl_2_O_3_ × FNCL_20_@CuO, which is attributed to defective oxygen-containing M–O bonds, and further confirms the presence of M–O.^26^ Since XPS mainly collects surface data, the M–O peak in γAl_2_O_3_ × FNCL_20_@CuO is less pronounced, because the surface is coated with γAl_2_O_3_.^16^ As illustrated in Fig. 2f, the XPS spectrum of Fe 2p reveals two prominent asymmetric peaks at 712.2 eV and 724.8 eV, which can be attributed to Fe 2p_3/2_ and Fe 2p_1/2_, respectively.^38^ The observed peaks at 711.4 eV and 715 eV are consistent with the presence of Fe(ii) and Fe(iii),^39^ respectively. Therefore, the observation of two peak positions at 711.04 and 712.5 eV can be attributed to the vibration peak of Fe^3+^,^27^ as depicted in Fig. S9. The minor peak corresponding to Fe^3+^, possibly originating from FNCL_20_@CuO during the oxidation reaction, was also detected in virgin γAl_2_O_3_ × FNCL_20_@CuO. The results suggest the successful incorporation of ions into the γAl_2_O_3_ × FNCL_20_@CuO framework.

Consequently, the Ni 2p spectra (Fig. 2g) exhibit two primary peaks, Ni 2p_3/2_ (856.9 eV) and Ni 2p_1/2_ (875.1 eV), accompanied by two satellite peaks.^40^ The Ni 2p_3/2_ and Ni 2p_1/2_ regions remained unchanged before and after the reaction, indicating that the chemical state of the nickel was well preserved.^41^ The Cu 2p peaks, which are typical of Cu^2+^ in CuO, are visible in Fig. 2h at 935.8 eV (Cu 2p_3/2_) and 954.9 eV (Cu 2p_1/2_). CuO is a widely used semiconductor and is widely known.^28^ To further elucidate the heterojunction formation and explore the synergistic interaction between metal double-layer hydrogen (FNCL) and CuO, as previously described by Ouyang *et al.*,^16^ is imperative. The high-resolution XPS spectra for Al, Fe, and Ni exhibited no substantial shift in peak positions following heterojunction formation, indicating that their valence states remain unaltered.

In Fig. 2i–k, the CV curves show that γAl_2_O_3_ × FNCL_20_@CuO has superior redox ability compared to γAl_2_O_3_, which is due to the extended potential window and increased current.^42,43^ Therefore, it can be inferred that the electrochemical process resulted in the disruption of the O–O bond and a rapid reduction in γAl_2_O_3_ on FNCL@CuO.^44,45^ In addition, the incorporation of FNCL@CuO into the structure (γAl_2_O_3_) has the potential to enhance the local concentration of Al ions during cycling, thereby promoting charge mobility and facilitating metal ion redox processes and charge transfer.^46^ Concurrently, the electron transfer capabilities of the catalysts (γAl_2_O_3_ and γAl_2_O_3_ × FNCL@CuO) are illustrated by EIS curves in Fig. 2l. The Randles equivalent circuits are instrumental in facilitating the simplification of several key components of the overall electrical circuit, including the resistance of the solution (*R*_s_), the double layer capacitance at the electrode surface (*C*_dI_), the charge transfer resistance (*R*_ct_), and the Warburg resistance(*Z*_w_)^47^ (Test S4). Thus, compared to γAl_2_O_3_, the arc radius in the EIS of γAl_2_O_3_ × FNCL@CuO exhibited an increase. The γAl_2_O_3_ catalyst exhibited a lower electron-transfer resistance and non-ideal capacitance behavior, indicating that doping with FNCL@CuO can facilitate the mutation of electrons at the interface. In the same way, the electrochemical impedance decreased as the density of γAl_2_O_3_ × FNCL@CuO sites increased. According to Hailian *et al.*,^48^ the γAl_2_O_3_ × FNCL@CuO catalyst demonstrates superiority in terms of activity over other comparable catalysts. This observation indicates that the judicious incorporation of a sufficient quantity of FNCL@CuO into the catalyst matrix can yield a greater number of active sites when compared to the use of a pure γAl_2_O_3_ catalyst.

### 3.3. 3D particle electrocatalytic performance evaluation

**Fig. 3:**
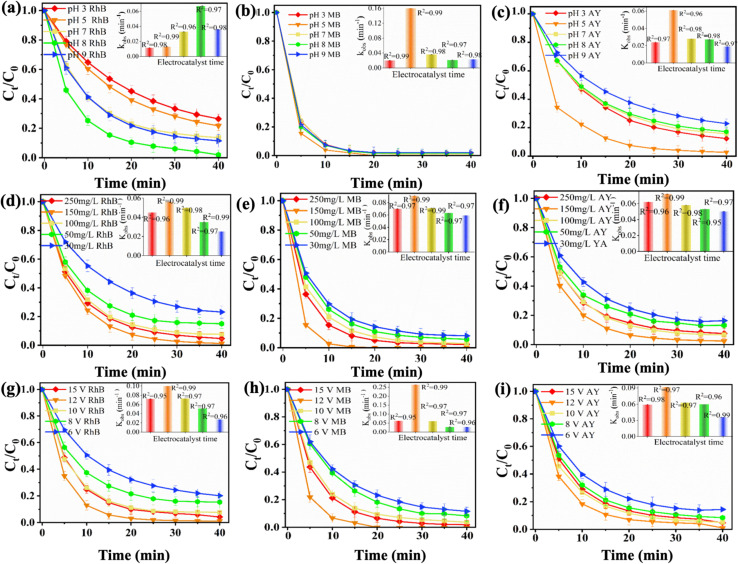
Degradation of organic pollutant using 3D system particle electrode γAl_2_O_3_ × FNCL_20_@CuO-400: (a) rhodamine B degradation in different pH, and the *k*_obs_(inset); (b) blue degradation in different pH, and the *k*_obs_(inset); and (c) acid yellow degradation in different pH, and the *k*_obs_(inset); (d) rhodamine B degradation in different mass, and the *k*_obs_(inset); (e) methyl blue, and the *k*_obs_(inset); and (f) acid yellow degradation in different pH, and the *k*_obs_(inset); (g) rhodamine B degradation in different voltage, and the *k*_obs_(inset); (h) methyl blue, and the *k*_obs_(inset); and (i) acid yellow degradation in different voltage (V), and the *k*_obs_(inset).

The effects of pH, intensity, and mass are crucial factors in the reaction mechanism of catalytic oxidation of organic pollutants using 3D particle electrodes. The pH level exerts a substantial influence on the electrolysis performance of the γAl_2_O_3_ × FNCL_20_@CuO-400 particle electrode. As the electrolysis duration increases, the RhB removal efficiency of the γAl_2_O_3_ × FNCL_20_@CuO-400 particle electrode progressively improves, as illustrated in Fig. 3a. At pH levels of 3, 5, 7, 8, and 9, the RhB removal efficiencies after 40 min are 73.5, 78.34, 86.40, 98.03, and 88.54%, respectively. A comparison of RhB removal efficiency by the γAl_2_O_3_ × FNCL_20_@CuO particle electrode under different pH values during the same electrolysis period shows that lower pH values result in reduced RhB removal efficiency. The RhB removal efficiency fluctuates significantly at higher pH values, with the most substantial improvement observed at pH 8, particularly under Baci conditions.

Fig. 3b demonstrates how the MB removal efficiency by the γAl_2_O_3_ × FNCL_20_@CuO-400 particle electrode progressively improves with increasing electrolysis time. The removal efficiencies at 25, 20, 20, 20, and 20 min are 98.23, 99.99, 99.88, 98.29, and 97.9% respectively, for pH levels of 3, 5, 7, 8, and 9. Notably, at pH 5 (acidic conditions), the MB removal efficiency increases compared to other pH values during the same electrolysis period. Under optimal conditions, the MB removal efficiency is lowest. This is because higher acid concentration and adjustment increase electrolyte density in water, thereby improving conductivity. Consequently, acid conditions are more favorable for enhancing MB removal by the γAl_2_O_3_ × FNCL_20_@CuO-400 particle electrode.^49^Fig. 3c illustrates the steady improvement of the AY removal capacity by the γAl_2_O_3_ × FNCL_20_@CuO-400 particle electrode with increasing electrolysis time. After 40 min at pH values of 3, 5, 7, 8, and 9, the corresponding AY removal efficiencies are 87.61, 97.25, 84.20, 82.78, and 77.10%, respectively. The results show that the order of electrolysis efficiency for the removal of AY is as follows: pH = 3, pH = 7, pH = 8, and pH = 9. The highest removal efficiency, 98.41%, is observed at pH 5. Furthermore, the presence of acidic conditions has been shown to promote the formation of AY precipitation, thereby enhancing its removal efficiency. This suggests that acidic environments may be more effective in this regard compared to alkaline environments.^50^ Thus, the removal of dyes (RhB, MB, and AY) from wastewater was performed using a 3D system operating at 12 V, 25 °C, and different dosages of γAl_2_O_3_ × FNCL_20_@CuO-400 particle electrodes as illustrated in Fig. 3d, the RhB concentration progressively decreased from 4.63 mg L^−1^ to 3.00, 1.55, 3.00, and finally, 0.25 mg L^−1^, as the particle electrode dosage increased from 50, 100, 150, and 150 mg L^−1^. However, when the particle electrode mass was further increased from 150 to 250 mg L^−1^, the RhB concentration rose slightly from 0.25 to 0.93 mg L^−1^.

As illustrated in Fig. 3e, the initial oxidation of MB was observed at an electrode dosage of 150 mg L^−1^, with complete degradation occurring within 20 min. At dosages of 30, 50, and 100 mg L^−1^, the MB concentration decreased progressively from 1.62 mg L^−1^ to 1.16 mg L^−1^ and 0.57 mg L^−1^, respectively. However, increasing the particle electrode mass from 150 mgL^−1^ to 250 mgL^−1^ resulted in a rise in MB concentration from 0.007 mg L^−1^ to 0.42 mg L^−1^. As illustrated in Fig. 3f, with an increase in the electrode concentration from 30 mg L^−1^ to 50, 100, and 150 mg L^−1^, the YA concentration gradually decreased from 3.27 mg L^−1^ to 2.76, 1.34, and 0.48 mg L^−1^, respectively. When the substrate electrode mass was increased from 50 mg to 250 mg L^−1^, the YA oxidation capacity increased from 0.48 mg L^−1^ to 1.51 mg L^−1^. These results indicate that as the dosage of the γAl_2_O_3_ × FNCL_20_@CuO-400 particle electrode increases, the number of active sites within the 3D reaction system also increases. This increase probably produced the formation of more hydroxyl radicals and thus increases the efficiency of the removal of RhB, MB, and AY. However, excessive buildup of particle electrodes within the reaction system may impede mass transfer during the reaction, which lowers the removal rates of RhB, MB, and AY as the particle electrode dosage increases.^24^ Furthermore, the enhanced degradation is primarily attributed to the destruction of the benzene ring structure in RhB, MB, and AY during the electrochemical reaction, leading to a gradual ring-opening and, in some cases, complete mineralization.^33^

The degradation of three organic pollutants, RhB, BM, and AY, from water, was studied using a bed particle electrode at 25 °C, with a γAl_2_O_3_ × FNCL_20_@CuO-400 dosage of 150 mg L^−1^, over different voltage levels (6, 8, 10, 12, and 15 V). Fig. 3g shows the effect of voltage on the oxidation of RhB at pH 6. The efficiency of the RhB elimination process gradually increases as the voltage rises from 6 V to 15 V. Maximum degradation (98.83%) from 12 V at 40 min, with a slight decrease to 98.1% observed at 15 V. The degradation of MB at different voltages and pH 4 is shown in Fig. 3h. The effectiveness of MB oxidation increases significantly at 12 V, reaching its peak between 12 V and 20 min. The elimination process progressively improves with voltage increments from 6 to 8 V, peaking at 40 min. Fig. 3i depicts the breakdown of AY at various voltages and pH 4. Increasing the voltage to 12 V and 15 V after 15 min enhances the early degradation process of AY. The removal efficiency continues to strengthen as the voltage increases, peaking between 12 V and 40 min. A minor decrease in oxidation was observed at 6, 8, 10, and 15 V. Therefore, it was determined that 12 V was the optimal voltage for the oxidation of RhB, MB, and AY from water using a 3D particle electrode (γAl_2_O_3_ × FNCL_20_@CuO-400). This voltage may correspond to the normal dihydrogen (H_2_) production potential, which activates hydrogen peroxide (H_2_O_2_), a key factor in degrading organic pollutants in wastewater. This finding suggests that higher currents can generate more ˙OH radicals in the reaction system, accelerating the pollutant removal rate.^51^ However, at elevated current levels, the electrolysis of water generates H_2_ and O_2_, which may impede further enhancement in degradation efficiency. As posited by Yaxin *et al.*,^24^ the O_2_ evolution reaction has the capacity to consume ˙OH radicals, thereby reducing their availability for degrading pollutants. The degradation rates of RhB and AY were 98.83% and 98.97% respectively, after 40 min, while the removal efficiency of MB reached 99.99% at 20 min, all at the same voltage of 12 V. Therefore, a summary of the removal efficiency of the metal-Al_2_O_3_ electrode from previous literature, along with the results from this study, the preparation of the new material is a simple and cost-effective process, and its application in treating pollutant dyes is straightforward. Furthermore, the degradation time of organic pollutants using the 3D system is faster than that reported in previous studies. This technology has the potential to serve as a more effective method for treating organic components in dyeing wastewater, with the previous study listed in Table S5.

### 3.4. Mechanism oxidation evaluation

**Fig. 4:**
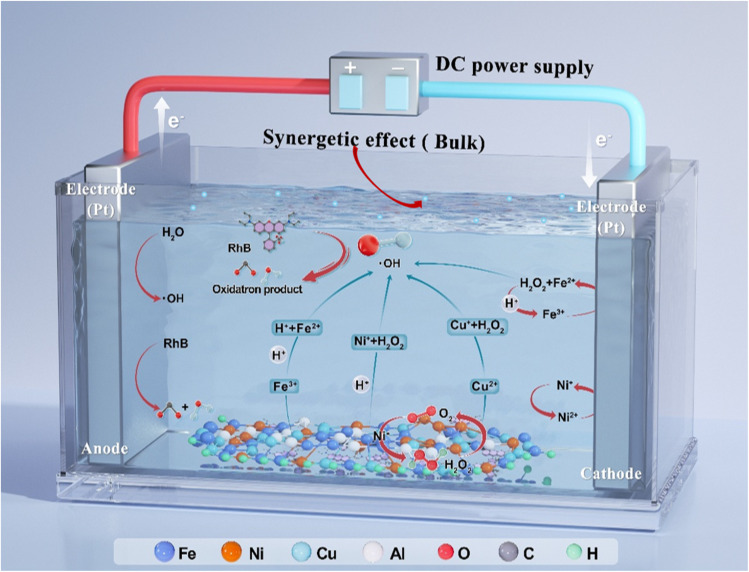
Mechanism of oxidation of organic pollutants.

The process for catalytic oxidation of organic pollutants (RhB, MB, and YA) over 3D porous catalysts is illustrated in Fig. 4, in conjunction with previously reported research. The 3D particle electrode system γAl_2_O_3_ × FNL_20_@CuO-400 can effectively treat refractory wastewater through synergistic reactions such as anode oxidation, particle electrode degradation, and homogeneous degradation.^52^ Before the pollutants' molecules are attacked by surface-active from the particle electrodes, they are first adsorbed onto active copper, Cu, Fe, Al, and Ni sites situated in the gaps between nanowire CuOx, Al_2_O_3_ × FNCLOx, Cu–Ox–Fe, Cu–Ox–Ni, and Cu–Ox–Al solid solutions.

Under the influence of Lewis acid sites, the oxidation of contaminants begins with the dissociation of the C–N bond through nucleophilic substitution. Free Cl^−^ ions are more likely to be adsorbed onto Brønsted acid sites, where they combine with H^+^ protons to form HCl. According to the literature, intermediates that may form during the oxidation of RhB in the 3D particle system include products from the cleavage of the C–N bond (such as C_26_H_33_O_3_N_2_), as shown in Table S6, products from the ring of carboxyl groups, a chain of aromatic compounds from the ring of the xanthene molecule, and naphthoquinone cleavage,^52^ as well as small aliphatic series compounds.^38^

Numerous investigations have revealed that in 3D particle electrode systems, the principal active species significant for the oxidation of resistant organic compounds is the ˙OH, as shown in eqn (1–6) (ref. 53) and eqn (7).^54^ Additionally, as previously noted, anodic oxidation and particle electrode oxidation occur when an electrolytic voltage is applied to the system. Based on the comparative results between different systems (2D and 3D), presented in Fig. S10 and Test S6, additional processes contributing to dye degradation can be inferred. The unique properties of a 3D porous structure accelerate adsorption and diffusion, increasing the contact area between reaction molecules and catalyst active sites. This leads to enhanced deep oxidation of pollutants, a reduction in the accumulation of heterogeneous catalytic reactions that produce H^+^ on the particle electrode surface, and the further exposure of additional active areas. Furthermore, γAl_2_O_3_ × FNCL_20_@CuO-400 exhibits a superior capacity for catalyzing H_2_O_2_ activation in the removal of pollutants. Additionally, the electrochemically generated H_2_O_2_ molecules adsorbed on particle electrodes have been shown to facilitate the reduction of Fe(iii) to Fe(ii), Ni(ii) to Ni(i), and Cu(ii) to Cu(i) through the 3D process. The subsequent oxidation of the generated Fe(ii), Ni(i), and Cu(i) ions on particle electrodes by H_2_O_2_ has been demonstrated to complete the Fe(iii)/Fe(ii) and Cu(ii)/Cu(i) redox processes, thereby producing ˙OH.^52,55^ Therefore, in three-dimensional systems, hydroxyl radicals have the capacity to directly attack the central carbon of RhB. This process results in the rapid decolorization of the solution due to the breakage of the dye molecules. In the course of this reaction, the principal products are benzene ring substances, including but not limited to benzoic acid, phthalic acid, and benzyloxy amine.^56^ As previously stated, the synergistic effect of γAl_2_O_3_ and FeNiCu ternary oxides has been demonstrated to significantly boost catalytic activity.^53^ Additionally, according to the previous literature, CuO induces more crystal defects, resulting in electron transfer in the 3D system. This system, introduced by the presence of Cu, Fe, Ni, and Al species, promotes the effective acceleration and activation of H_2_O_2_. Specifically, the four redox couples Cu^2+^/Cu^+^, Ni^2+^/Ni^3+^, Fe^2+^/Fe^3+^, as shown in eqn (7–11),^53^ and Al^3+^/Al^2+^, facilitate good electron transfer and consistency at the Cu–Ox–Fe, Cu–Ox–Ni, and Cu–Ox–Al interfaces.

1

<svg xmlns="http://www.w3.org/2000/svg" version="1.0" width="23.636364pt" height="16.000000pt" viewBox="0 0 23.636364 16.000000" preserveAspectRatio="xMidYMid meet"><metadata>
Created by potrace 1.16, written by Peter Selinger 2001-2019
</metadata><g transform="translate(1.000000,15.000000) scale(0.015909,-0.015909)" fill="currentColor" stroke="none"><path d="M80 600 l0 -40 600 0 600 0 0 40 0 40 -600 0 -600 0 0 -40z M80 440 l0 -40 600 0 600 0 0 40 0 40 -600 0 -600 0 0 -40z M80 280 l0 -40 600 0 600 0 0 40 0 40 -600 0 -600 0 0 -40z"/></g></svg>


Fe(ii) + 2H^+^ → Fe^2+^ + H_2_

2 Fe^2+^ + H_2_O_2_ → Fe^3+^ + ˙OH + OH^−^

3 Cu(ii) + 2H^+^ →  Cu^2+^ + H_2_

4 Cu^2+^ + e^−^ → Cu^+^

5 Cu^2+^ + H_2_O_2_ → Cu^+^ + ˙HO_2_ + H^+^

6 Cu^+^ + H_2_O_2_ → Cu^2+^ + ˙OH + OH^−^

7 R + ˙OH → oxidated products

8 ≡Fe(iii) + H_2_O_2_ → ≡Fe(ii)+ ˙HO_2_ + H^+^

9 ≡Fe(ii) + H_2_O_2_ → ≡Fe(iii) + ˙OH + OH^−^

10 ≡Cu(ii) + H_2_O_2_ → ≡Cu(i)+ ˙HO_2_ + H^+^

11 ≡Cu(i) + H_2_O_2_ → ≡Cu(ii) + ˙OH + OH^−^

**Fig. 5:**
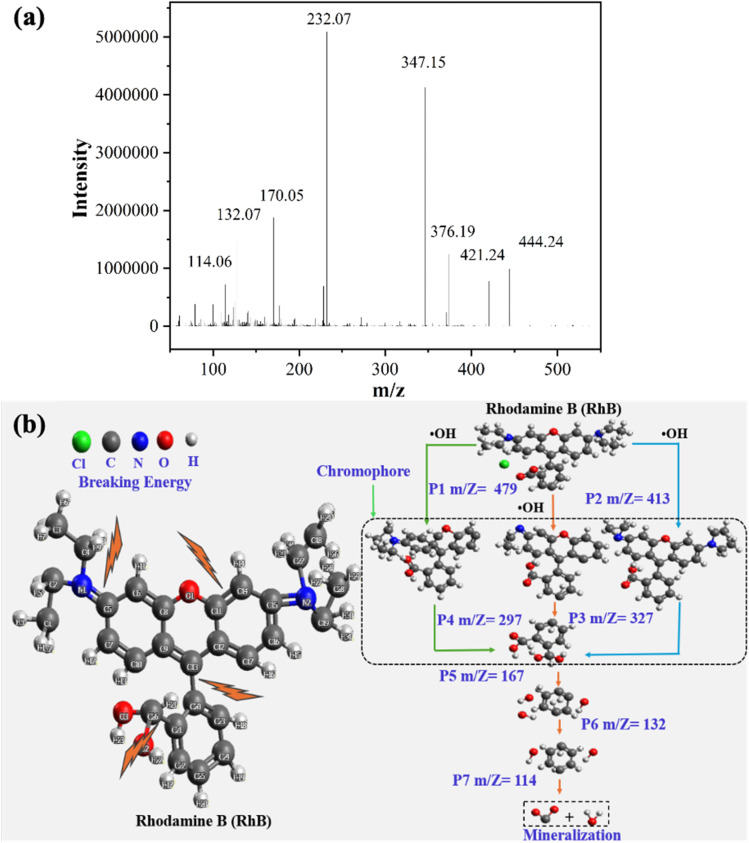
(a) Liquid chromatography mass spectra (LC-MS) of the intermediates at 20 min oxidation in the 3D system, and (b) degradation pathway of RhB.

Finally, a possible degradation pathway of RhB, MB, and AY is proposed in Fig. 5 and Table S6. At the outset, RhB undergoes an initial reaction with ˙OH radicals, leading to the dehydrogenation of RhB, which produces P1 and H_2_O. For example, the use of LC-MS to examine the degradation products has facilitated a more comprehensive investigation into the RhB oxidation process. The data set revealed the presence of product peaks with molecular weights lower than that of RhB, including the most significant mass-to-charge ratios (*m*/*z*) as illustrated in Fig. 5a. Concurrently, the molecular weight products were identified at *m*/*z* values was 114.06, 132.07, 170.05, 375.18, 421.24, and 444.24. Thus, the total ion chromatograms for negative and positive ions of RhB are shown in Fig. S11 and S12. The integrated peak results and the raw mass spectra data corresponding to each peak are provided in the attachment. However, the result of the molecular weight of the negative and positive ions, likely RhB oxidation intermediates, varied listed in Table S8.

According to previous research, the hydrogen atom in the aromatic ring is less likely to be extracted by ˙OH.^57^ The results of the investigation demonstrate that a substantial amount of ˙OH is produced during the process of degradation.^58^ As the Fig. 5b, the degradation of RhB occurs through a series of reactions, including *N*-deethylation, deamination, dealkylation, decarboxylation, chromophore splitting, ring opening, and mineralization.^59^ The degradation of RhB (*m*/*z* = 443) commences with the *N*-deethylation of C=N, progressing sequentially to yield *N*,*N*-diethyl-*N*′-ethyl rhodamine (*m*/*z* = 327 and *m*/*z* = 298).^60^ As Boge *et al.* assert, *N*-ethyl is a color-assisting group with an auxiliary color effect.^52^ However, the observed decoloration of RhB was primarily attributed to the cleavage of the chromophore conjugated group structure. Concurrently, the destruction of the dye molecule conjugate system transpires, thereby enabling the hydroxyl radical to directly attack the RhB center carbon. Consequently, the dye undergoes rapid decolorization. The *N*-ethyl group was found to be susceptible to the effects of the conjugated system, and the hydroxyl radical^56^ efficiently facilitated its removal. The hydroxyl radical persists in its attack on the intermediate with a *m*/*z* value of 327, thereby facilitating the deamination and demethylation processes.

These reactions can yield the intermediate products with *m*/*z* values of 167, 132, and 114, respectively. Concurrently, the chromophore structure may undergo cracking, resulting in the formation of monocyclic aromatic compounds.^61^ The products resulting from the cleavage of carboxyl include a series of aromatic compounds, which are generated from the cleavage of the xanthene molecule group and naphthoquinone ring, as well as some small aliphatic chain compounds.^52^ Finally, low molecular weight organic acids or amides are subjected to further mineralization, resulting in the formation of inorganic products,^60,62^ with the concomitant release of H_2_O and CO_2_.^63^ The findings from this research may provide valuable insights into developing enhanced processes and particle electrode designs for three-dimensional systems.

## 4. Exploring the impact of anions, water matrices, stability, and quenching experiments on the 3D electrode reaction

**Fig. 6:**
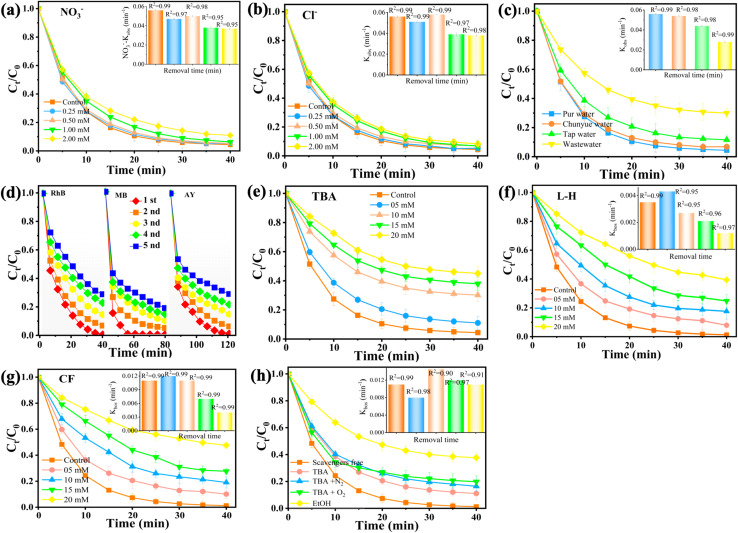
Effect of anions (a) NO_3_^−^, (b) Cl^−^, (c) real water matrices, (d) stability and quenching experiments (e) *tert*-butyl alcohol (TBA), (f) l-histidine (l-H), (g) chloroform (CF), and (h) effect of different scavengers on RhB degradation in 3D particle electrode γAl_2_O_3_L_2_ × FNC_0_@CuO-400 process. Conditions: [catalyst]_0_ = 150 mg L^−1^, [RhB]_0_ = 20 mg L^−1^, pH = 8, and 12 V, *T* = 25 °C.

On the γAl_2_O_3_ × FNCL_20_@CuO-400 surface, the alkaline solution promotes the formation of inactive nanowire CuO@FNC hydroxides, which reduces the catalytic activity. Scavengers free radical (ascorbic acid), *tert*-butyl alcohol (TBA), l-histidine (l-H), CF, EtOH, and anions such as NO_3_^−^, Cl^−^, and HCO_3_^−^/CO_3_^2−^, and a water matrix can impact the oxidation of dye pollutants in the 3D electrode reaction. The quenching experiment condition was illustrated in Test S5. As shown in Fig. 6a, NO_3_^−^ had no discernible effect on RhB oxidation, and even when the NO_3_^−^ dosage was adjusted from 0.25 to 2.0 mM, the RhB oxidation capacity was maintained at 95.59%. This may indicate that the above-mentioned interference by background ions can be effectively countered^64^ by the 3D particle electrode process (γAl_2_O_3_ × FNCL_20_@CuO-400). As shown in Fig. S13, HCO_3_^−^/CO_3_^2−^ hurts RhB oxidation, and C^−^ has a small negative effect on RhB degradation, as shown in Fig. 6b. On the γAl_2_O_3_ × FNCL_20_@CuO-400 surface, the high pH promotes the formation of inactive nanowire-CuO, double-layer FeNi with hydroxides, which reduces the catalytic activity.^65^ This suggests that the aforementioned interference by background ions can be effectively countered by the 3D particle electrode technique (γAl_2_O_3_ × FNCL_20_@CuO-400). Then, the degradation of RhB was further investigated in wastewater (WW), tap water (TW), pure water (PW), and Chunyue water (CW). Compared to deionized water, the degradation of RhB in CW decreased only slightly by 3.20%, as shown in Fig. 6c. In TW and WW samples, RhB was much more inhibited, and after 40 min, RhB degradation reduced to 91.1% and 87.2%, respectively. A more complex composition of the water matrix makes the target pollutants more competitive, leading to a further noticeable electron influence on RhB oxidation in the three-dimensional system.

The stability and reusability of the particle electrode γAl_2_O_3_ × FNCL_20_@CuO-400 were tested, and the results are illustrated in Fig. 3d. After the RhB doxiadation reaction, γAl_2_O_3_ × FNCL_20_@CuO-400 was filtered and purified for further use to assess its stability. During the first cycle, RhB was fully degraded within 40 min.^53^ The RhB removal rates slightly decreased in the subsequent cycles, with efficiencies of 98.83%, 84.99%, 76.76%, and 70.74% for the second, third, fourth, and fifth cycles, respectively. The stability and reuse test for MB followed the same procedure. The MB removal efficiency following the initial cycle was 99.99%, yet it underwent a decline to 90.54%, 81.63%, and 85.99% in the subsequent cycles. Furthermore, γAl_2_O_3_ × FNCL_20_@CuO-400 was examined in succession for the elimination of AY across the second, third, fourth, and fifth cycles, yielding removal rates of 98.97%, 84.77%, 77.95%, and 70.57%, respectively. Even after five consecutive cycles, γAl_2_O_3_ × FNCL_20_@CuO-400 demonstrated a removal rate of more than 70% for RhB, MB, and YA, indicating excellent recycling stability.^43^ A comparative analysis of the reusability of γAl_2_O_3_ × FNCL_20_@CuO-400 reveals that it is comparable to, or even superior to, other materials reported in the literature. These findings suggest that γAl_2_O_3_ × FNCL_20_@CuO-400 has the potential to serve as an effective particle for 3D systems. The observed decrease in performance may be attributed to the loss of Fe^2+^, N^+^, and Cu^2+^ during the technology process, which could reduce the particle capacity of the electrode.

Quenching tests were examined to evaluate the role of various reactive species in the 3D particle electrode γAl_2_O_3_ × FNCL_20_@CuO-400 process. Crucially, the hydroxyl in TBA (Fig. 6e) can interact with Al, Fe, Ni, and Cu in the γAl_2_O_3_ × FNCL_20_@CuO-400 particles to form complexes that could reduce the availability^65^ of the catalytically active site of the iron-nickel hydroxide bilayer of nanowire-CuO. Therefore, a reasonable role for ^1^O_2_ in the γAl_2_O_3_ × FNCL_20_@CuO-400 process is suggested by the significant marginal influence that histidine (l-H) showed on RhB degradation when added at a higher concentration (Fig. 6f). According to the findings above, in the 3D particle electrode γAl_2_O_3_ × FNCL_20_@CuO-400 process, ˙OH and ^1^O_2_ are not considered important reactive species for RhB degradation. Thus, a modest participation of O_2_˙^−^ in the γAl_2_O_3_ × FNCL_20_@CuO-400 process is suggested by the considerable marginal influence of chloroform (CF) on RhB degradation as seen in Fig. 6g when it was introduced at a higher concentration. The results indicate that ˙OH and O_2_˙^−^ are exempted as critical reactive principal for RhB oxidation in the 3D particle electrode γAl_2_O_3_ × FNCL_20_@CuO-400 process.^65^ For ˙OH, EtOH, and TBA were used as the scavengers. RhB degradation efficiencies decreased from 98.82% to 3.02%, 9.87%, 13.07%, 14.82%, and 29.08% with inhibition rates of 95.80%, 88.95%, 85.73%, 84%, and 69.8%, respectively, following the addition of scavenger free radical (ascorbic acid), EtOH, TBA, TBA + N_2_, and TBA + O_2_, as illustrated in Fig. 6h. This suggests that ˙OH contributed more to RhB degradation. Thus, ˙OH steady-state concentrations were measured quantitatively. The findings mentioned above showed that during the activation of the 3D system, ^1^O_2_, O_2_˙^−^, and ˙OH were generated and that ˙OH was crucial to the degradation of RhB. EtOH successfully prevented RhB degradation, as illustrated in Fig. 6h, whereas TBA did not affect RhB degradation, indicating that ˙OH was insignificant in the 3D particle electrode γAl_2_O_3_ × FNCL_20_@CuO-400 process. The probes *p*-chlorobenzoic acid and nitrobenzene, which demonstrated poor reactivity towards ^1^O_2_ and R–O˙, were used to assess ˙OH.^65,66^ The reasonable contribution of in ˙OH the 3D particle electrode v processes is further supported by Fig. S14, which shows that the oxidation performance of p-CBA and NB is low after 40 min and that the addition of TBA effectively suppresses their oxidation. The results of the radical scavenger degradation suggest that the γAl_2_O_3_ × FNCL_20_@CuO-400 particles with an interconnected hierarchical porous structure may be oxygen vacancies (VOs), which can be successfully prepared by a simple complexation and solvothermal process method followed by a calcination method. Therefore, more in-depth studies are needed to confirm that the VOs improve catalyst performance in 3D systems.

## 5. Conclusion

This study demonstrates the successful fabrication of a 3D γAl_2_O_3_ × FNCL@CuO particle electrode under optimized synthesis conditions. Comprehensive characterization confirmed the effective loading of CuO nanowires and FeNi-LDH, which enhanced charge transfer and catalytic activity. The electrode achieved high removal efficiencies of 98.82% for RhB, 99.99% for AY, and 98.97% for MB, significantly outperforming pure γAl_2_O_3_. The porous 3D structure was shown to accelerate oxidation by increasing the interface between active sites and reactants. Quenching experiments identified radical species as the primary active agents, and the electrode maintained stable performance over five consecutive cycles. These results confirm the material's potential for efficient and durable electrochemical wastewater treatment.

## Author contributions

Guene L Razack: conceptualization, methodology, formal analysis, writing – review & editing. Jie Ding: investigation, data curation. Xian Zhao: writing – review & editing. Ya-Ni Zang: resources, supervision. Chen-Hao Cui: investigation, software. Wen-Shuo Wang: project administration. Ji-Wei Pang: writing – review & editing. Lu-Yan Zhang, Nan-Qi Ren, Worou Chabi Noel, Assogba Dou Rached: writing – review & editing. Shan-Shan Yang: formal analysis, visualization, writing – original draft, writing – review & editing.

## Conflicts of interest

The authors certify that no competing financial interests or personal relationships have influenced the work presented in this study.

## Supplementary Material

RA-016-D5RA06641G-s001

## Data Availability

The data are available on request from the authors. The data that support the findings of this study are available from the corresponding author upon reasonable request. Supplementary information (SI) is available. See DOI: https://doi.org/10.1039/d5ra06641g.
