# Supplementary material for: Design of a 3D nanowire-CuO/LDH@FeNi-γAl2O3 catalyst and its synergistic mechanism for accelerated dye degradation in wastewater
Source: RSC Adv. 2026 Jan 19;16(2):1682–96. doi: 10.1039/d5ra06641g (PMC12814818; doi:10.1039/d5ra06641g)
Supplement: RA-016-D5RA06641G-s001 [file RA-016-D5RA06641G-s001.pdf]

Supplementary Information (SI) for RSC Advances.
This journal is © The Royal Society of Chemistry 2026

Supporting information

Design of a 3D Nanowire-CuO/LDH@FeNi-γAl_2O_3 Catalyst and Its Synergistic Mechanism for Accelerated Dye Degradation in Wastewater

Guene L. Razack[a,b,c,d], Jie Ding[a,b,*], Xian Zhao[a,b], Ya-Ni Zang[a,b], Chen-Hao Cui[a,b], Wen-Shuo Wang[a], Ji-Wei Pang[e], Lu-Yan Zhang[f], Worou Chabi Noel[c,d], Assogba Dou Rached[c,d], Nan-Qi Ren[a,b], Shan-Shan Yang[a,b,*]

[a]*National Engineering Research Center for Safe Disposal and Resources Recovery of Sludge, Harbin Institute of Technology, Harbin 150090, China*

[b]*State Key Laboratory of Urban-rural Water Resource and Environment, School of Environment, Harbin Institute of Technology, Harbin 150090, China*

[c]*Laboratory of Materials and Structures (LAMS). Graduate School of Civil Engineering Véréchaguine, 02 BP 244 Cotonou, Benin*

[d]*Laboratory of Water Science and Technology, National Water Institute (INE), University of Abomey-Calavi, 01 BP 526 Cotonou, Benin.*

[e]*Harbin Corner Science & Technology Inc., Harbin 150023, China*

[f]*School of Environmental Science and Engineering, Yancheng Institute of Technology, Yancheng 224051, China*

**Corresponding authors: Shan-Shan Yang, shanshanyang@hit.edu.cn; Jie Ding, dingjie123@hit.edu.cn.*

Test S1. Chemicals and Materials

The copper foam was sourced from Kunshan Jiayixing Electronic Technology Co., LTD. γAl_2O_3 was obtained from Shanghai Yaoyi Alloy Material Co., LTD. Sodium Hydroxide (NaOH, 100%), and ammonium Persulfate $(NH_4)_2S_2O_8$, 99%) were provided by Tianjin Tianli reagent Co., LTD. Nickel nitrate hexahydrate ($Ni(NO_3)_2·6H_2O$, 98%) was supplied by Chemical Reagent Co, LTD. Ferrous Sulphate ($FeSO_4·7H_2O$, ≥ 99%) was acquired from Tianjin BASF Chemical Co., LTD. Additionally, hydrochloric acid (HCl, 33%), acetone, and anhydrous ethanol (EtOH, ≥ 99.8%), were obtained from Macklin Reagent Co., LTD. Rhodamine B (RhB) 95% was supplied by MIKA-Aiochemika Reagent Co., LTD, with acid yellow (AY) 36 and methyl blue (MB) provided by Sigma-Aldrich Co., LTD. N, N-dimethylformamide ($HCON(CH_3)_2$, 99.5%) from Tianyang Tianli Chemical Reagent Co., Ltd, Sodium carbonate (Na_2CO_3), sodium chloride (NaCl, ≥ 99.5%), Tianjin Tianli Chemical Reagent Co., Ltd, sodium bicarbonate ($NaHCO_3$), Sodium nitrate ($NaNO_3$), Tianjin Yongda Chemical Reagent Co., Ltd, chloroform (CF, ≥ 99.0%), L-Histidine (L-H), and tert-butanol (TBA) were purchased from Sinopharm Chemical Reagent Co., Ltd. (Shanghai, China). Deionized water, used in all experiments, was obtained from a Milli-Q academic system. All the chemicals were analytical grade without further purification.

Table S1 Several chemical names and abbreviations

Chemical	Nam	Abbreviation
γAl_2O_3	Alamina	γAl_2O_3
Fe	iron	F
Ni	Nickel	N
Cu	Copper	C
O	Oxygen	O
LDH	Double-layer hydrogen	L
γAl_2O_3xFeNiCu-LDH@CuO	Material	γAl_2O_3xFNCL@CuO

Test S2. Methods and calculations of the performance removal rate analysis

The removal rate of RhB, MB, and AY, noted as XRhB, XMB, and XAY, was defined according to Eq. (S1-S3) respectively.

$$x_{RhB} = \frac{[RhB]_0 - [RhB]t}{[RhB]_0} * 100\% \qquad \text{(S1)}$$

$$x_{MB} = \frac{[MB]_0 - [MB]t}{[MB]_0} * 100\% \qquad \text{(S2)}$$

$$x_{YA} = \frac{[YA]_0 - [AY]_t}{[AY]_0} * 100\% \qquad \text{(S3)}$$

In the context of dye wastewater treatment, X_{RhB}, X_{MB}, and X_{AY} denote the removal efficiency of RhB, MB, and AY, respectively. The variables $[RhB]_0$, $[MB]_0$, and $[AY]_0$ represent the initial concentrations of RhB, MB, and AY in the coking wastewater, whereas $[RhB]_t$, $[MB]_t$, and $[AY]_t$ refer to the residual concentrations of RhB, MB, and AY, respectively, following the 3D particle electrode treatment. Subsequently, the residual concentration of Rhodamine B, methyl blue, and yellow acid in the solution was determined using a TU-1901 UV-vis spectrophotometer at a wavelength of 550 nm, 650 nm, and 435 nm through colorimetric analysis. The values of UV_{550}, UV_{650}, and UV_{435} were determined using the following equations: S(4-6):

$$RhB{:}UV_{570} = \frac{A}{b} * C \qquad \text{(S4)}$$

$$MB{:}UV_{600} = \frac{A}{b} * C \qquad \text{(S5)}$$

$$YA{:}UV_{435} = \frac{A}{b} * C \qquad \text{(S6)}$$

In this equation, C_t (mg/L) denotes the RhB, MB, or AY concentration of the dye wastewater at a given time, t. C_0 (mg L^{-1}) represents the initial concentration of RhB, MB, or AY in the dye wastewater. The parameter k is the pseudo-first-order rate constant, expressed in minutes per min Eq. (S7):

$$\ln C_t = -kt + \ln C_0 \tag{S7}$$

where Ct (mg/L) is the RhB, MB, or AY concentration of the dye wastewater at time t; C_0 (mg L^{-1}) is the RhB, MB, or AY concentration of the dye wastewater at the initial time; and k is the pseudo-first-order rate constant (min^{-1}).

Test S3. CV and EIS electrocatalysts test sample preparation

The electrode materials are evaluated for their electrochemical properties using the CHI760E electrochemical working station. In the course of each electrochemical evaluation, encompassing both cyclic voltammetry (CV) and electrochemical impedance spectroscopy (EIS), the electrolyte, counter electrode, and reference electrode were consistently established as 5 M KOH aqueous solution, Pt wire, and Hg/HgO electrode, respectively. The substance contained within the electrode system has a loading mass of approximately 7 mg. The utilized electrolyte was 5 M KOH.

The three-electrode system is utilized in the test. The reference electrode employed is Hg/HgO, and the counter electrode is platinum.

The EIS test was conducted within the frequency range of 100 kHz to 0.1 Hz, with an amplitude ranging from 10 to 40 mV.

Test S4. EIS electroanalytical methods

According to Uygun et al.[1], the electron-injection system (EIS) circuit and the redox reaction occur at the surface of the working electrodes in a conventional electrochemical cell, otherwise known as a three-electrode system. R_{ct} is an abbreviation for "charge transfer resistance," Rs is an abbreviation for "electrolyte resistance," and C_{dt} is an abbreviation for "capacitance double layer."

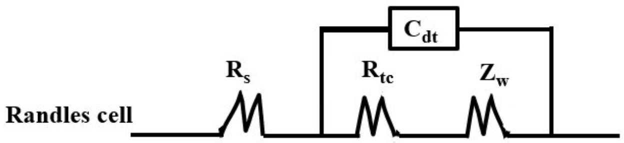

Fig. S1. The scheme is described as straightforward of the EIS circuit and the redox reaction that occurs at the surface of working electrodes in a traditional electrochemical cell.

Equation S8 quantifies the correlation between the applied frequency (f) and the radial frequency (ω).

$$w = 2 \cdot \pi \cdot f \qquad \text{(S8)}$$

In a linear system, the signal undergoes a phase shift (Φ) and exhibits a variation in amplitude relative to I_0 (Equation S9).

$$I_t = I_0 \sin (wt + \Phi) \qquad \text{(S9)}$$

Therefore, as indicated by Equation S10, the impedance of the entire system can be calculated. Substituting z for E and I, we obtain the following equation:

$$z = \frac{E}{I} = z_0 \exp i\Phi = z_0(\cos \Phi + i \sin \Phi) \qquad \text{(S10)}$$

The variables Z, E, I, ω, and Φ represent impedance, potential, current, frequency, and phase shifts, respectively, between E and I.

Test S5. Quenching information

The quenching experiment is analogous to the oxidation experiment of RhB, with the salient difference being the simultaneous addition of quenching, an anion molecule, and an organic compound. This method has been employed by Jinxiang et al.[2]. Quenching experiments were performed with the addition of NO_3^-, Cl^-, HCO_3^-/CO_3^-, and TBA, which were employed as scavengers for nitrate radicals (NO_3^-), chlorine radical (Cl^-), carbonate radical (CO_3^{2-}), and organic TBA, respectively. The quenching experiment was conducted at room temperature in 250-mL clear glass beakers, with the stirring rate maintained at a constant level. Subsequently, 150 mg/L of $\gamma Al_2O_3xFNCL_{20}$@CuO-400 catalysts were added to a solution containing 20 mg/ L RhB, and the mixture was stirred. The pH value of the solutions was then adjusted to 8.0. The initiation of the reaction was facilitated by the incorporation of 0.25, 0.50, 1, and 2 mM quenchers, along with 12 V, within a 3D system. Samples were collected and quenched after reacting for a specific time. Subsequently, the solution was filtered with a 0.22-micrometer membrane to remove the catalysts before analysis.

2. Removal efficiency of the RhB, MB, and AY using the different catalysts

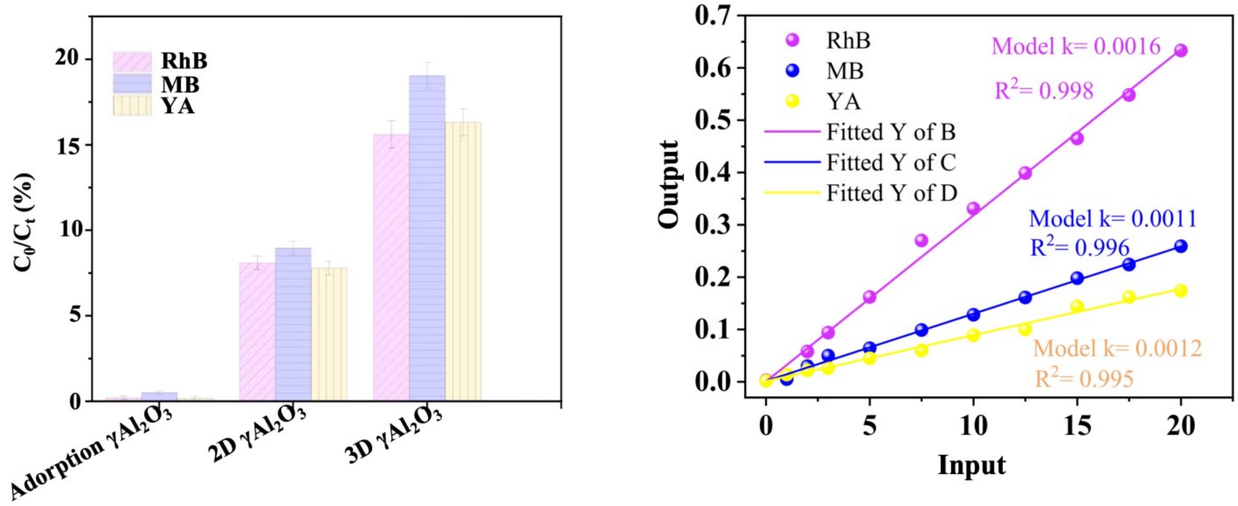

Fig. S2. Performance removal of RhB, MB, and AY using γAl_2O_3 catalytic, initial condition: 20 mg L^{-1} of RhB, MB, and YA, 12V, at lab temperature.

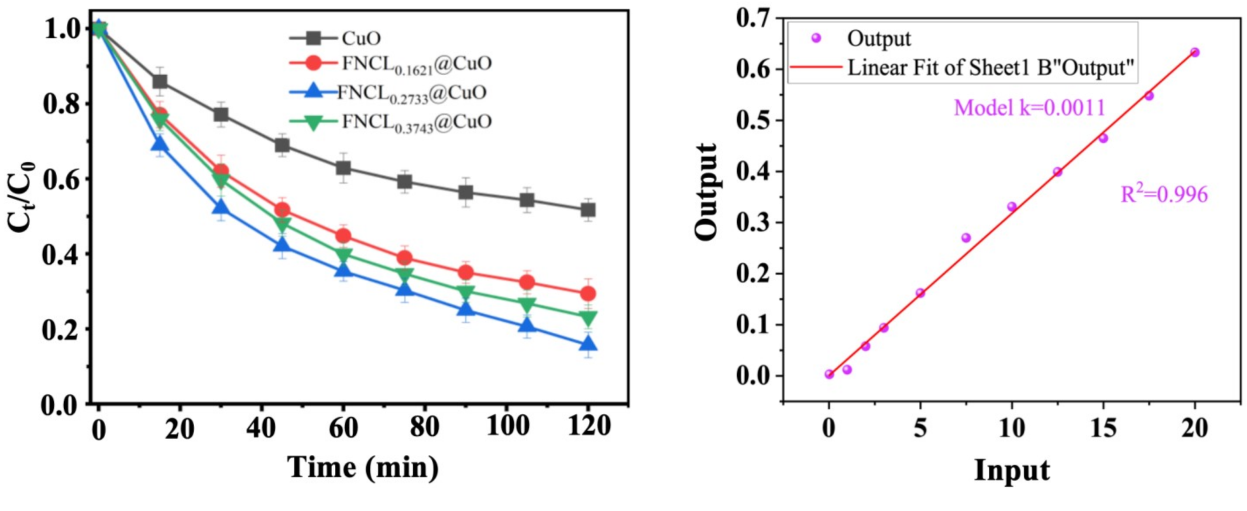

Fig. S3. performance of the different catalytic FeNiCu-LDH@CuO-0.1621, FNCL@CuO-0.2733, and FNCL@CuO-0.3742 using a 3D electrode to remove RhB, MB, and AY, initial condition: 20 mg L^{-1} of RhB, 12V, at lab temperature.

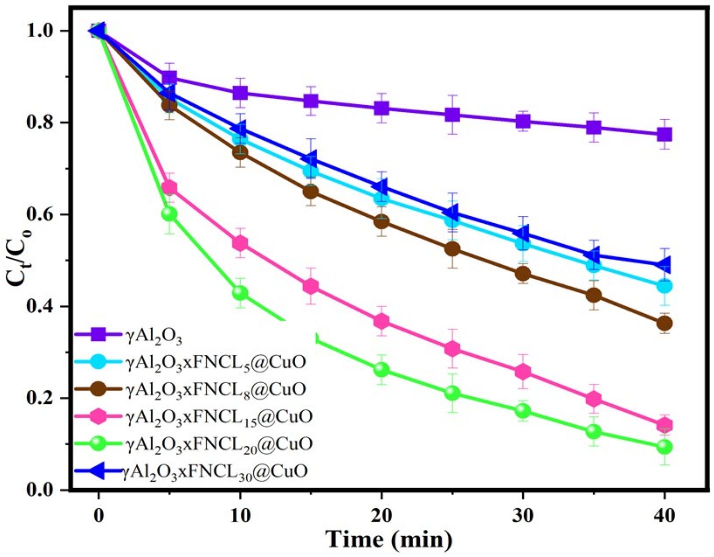

Fig. S4. The removal performance of the different concentrations γAl_2O_3xFNCL@CuO, initial condition: 20 mg L^{-1} of RhB, 12 V, at lab temperature

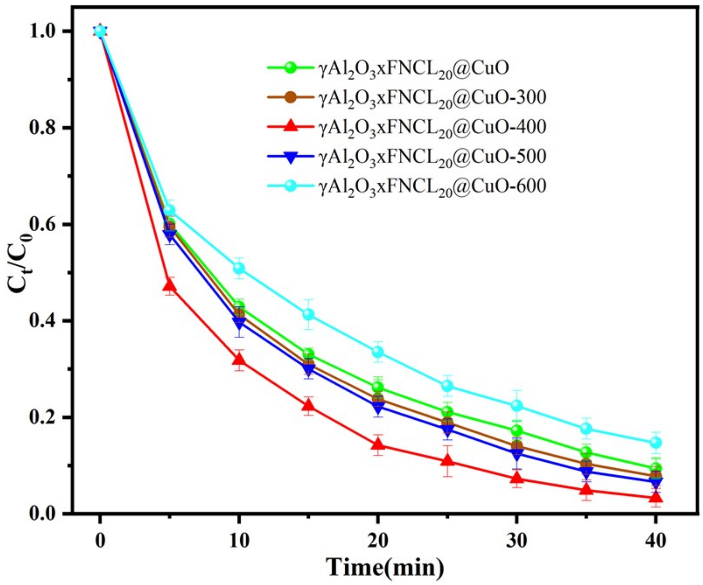

Fig. S5. The calcination removal performance of RhB from wastewater using the 3D system, initial condition: 20 mg L^{-1} of RhB, 12 V, at lab temperature

Table S2 The performance removal of the catalysts in the 3D system.

Materials	system	Removal rate (%)		
		RhB	MB	AY
γAl_2O_3	Adsorption	8.08	8.95	7.78
	Without γAl_2O_3	10.53	12.82	10.62
	3D	15.6	19.03	16.3
$FNCL_{0.1621}$@CuO	3D	70.6	-	-
$FNCL_{0.2733}$@CuO	3D	84.27	-	-
$FNCL_{0.3742}$@CuO	3D	76.8	-	-
$\gamma Al_2O_3xFNCL_5$@CuO	3D	55.55	-	-
γAl_2O_3xFNCL8@CuO	3D	63.7	-	-
$\gamma Al_2O_3xFNCL_{15}$@CuO	3D	85.85	-	-
$\gamma Al_2O_3xFNCL_{20}$@CuO	3D	90.6	-	-
$\gamma Al_2O_3xFNCL_{30}$@CuO	3D	50.96	-	-
$\gamma Al_2O_3xFNCL_{20}$@CuO-300	3D	79.52	-	-
$\gamma Al_2O_3xFNCL_{20}$@CuO-400	3D	96.69	-	-
$\gamma Al_2O_3xFNCL_{20}$@CuO-500	3D	88.38	-	-
$\gamma Al_2O_3xFNCL_{20}$@CuO-600	3D	85.30	-	-

3. Characterization of the new particle electrode synthesized

Table S3 Physical parameters of catalysts

Catalysts	Al (At. %)	O (At. %)	Fe (At. %)	Ni (At. %)	Cu (At. %)
γAl_2O_3	56	64	N. A[a]	N. A[a]	N. A[a]
$FNCL_{20}$@CuO	N. A	16.3	38.5	27.5	18.7
γAl_2O_3xFNCL@CuO-400	41.8	47.2	5.9	2.8	2.3

Note: [a] Not addicted

Table S4 Physical parameters and lattice parameters of catalysts

Catalysts	Pore volume ($cm^3.g^{-1}$)	S_{BET} ($m^2.g^{-1}$)	Average pore diameter (nm)	Lattice parameters

				(%)
γAl_2O_3	0.11	15	12.4	8.5
γAl_2O_3xFNCL@CuO-400	0.99	127	7.8	8.2

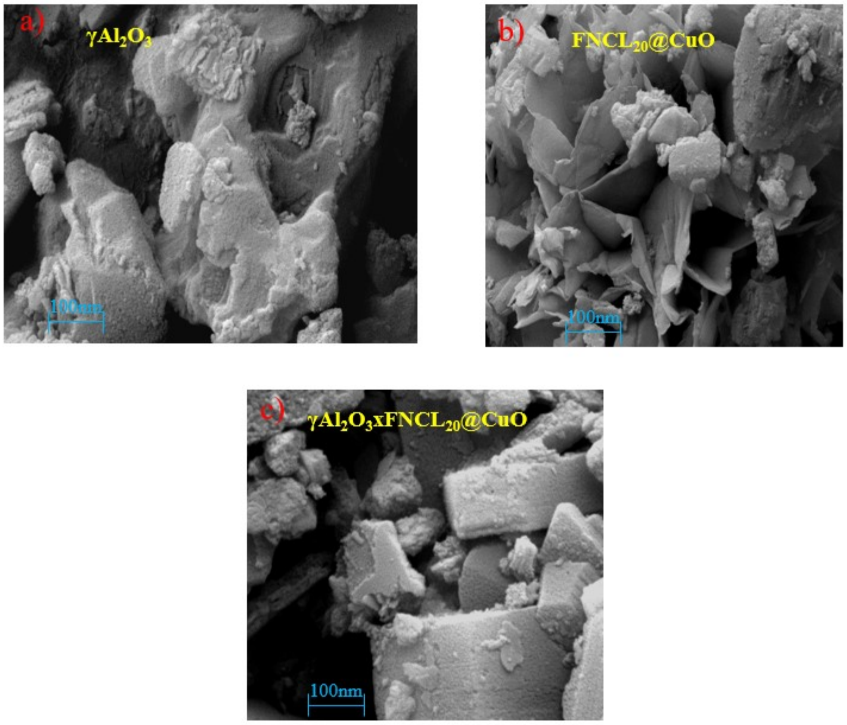

Fig. S6. Scanning electron microscope (SEM) images at 100 nm of the a) γAl_2O_3: b) FNCL@CuO, c) γAl_2O_3x$FNCL_{20}$@CuO.

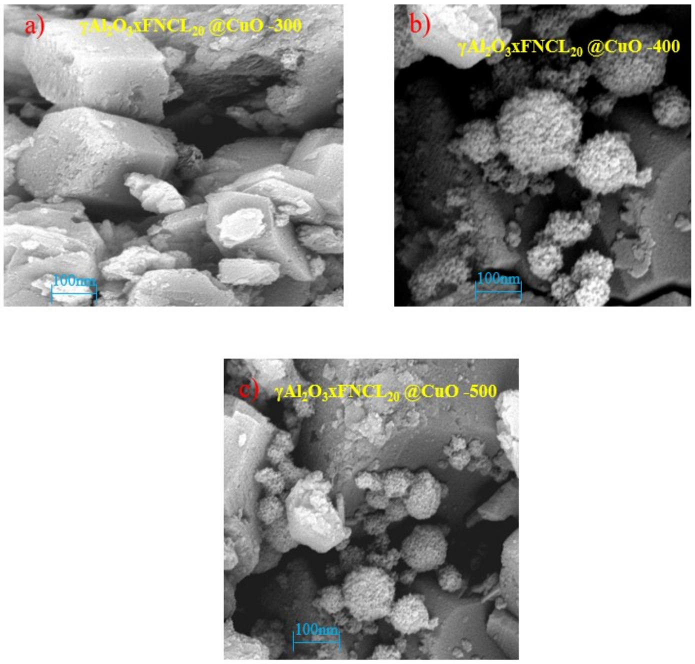

Fig. S7. Scanning electron microscope (SEM) images at 100 nm of the different calcination: a) $\gamma Al_2O_3xFNCL_{20}$@CuO-300, b) $\gamma Al_2O_3xFNCL_{20}$@CuO-400, c) $\gamma Al_2O_3xFNCL_{20}$@CuO-500.

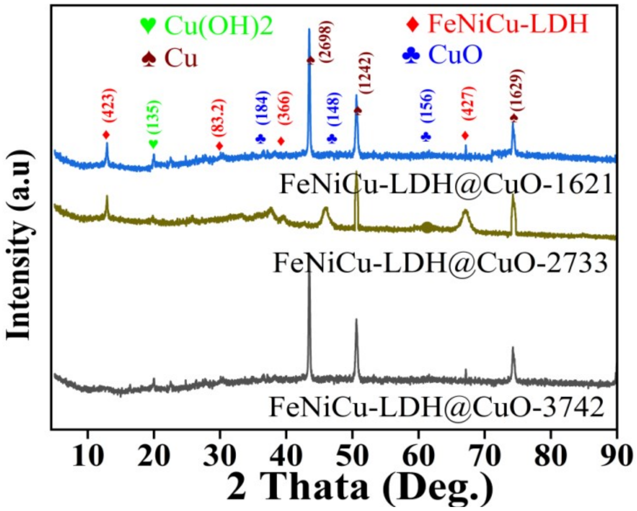

Fig. S8. XRD of the FeNiCu-LDH@CuO particle electrode

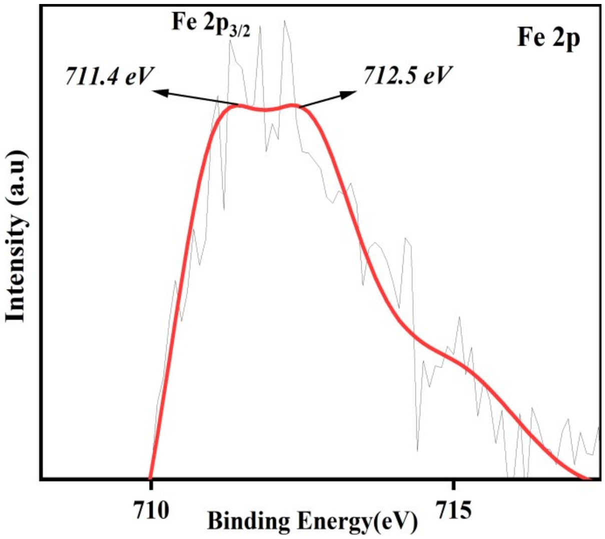

Fig. S9. Characterization XPS (Fe 2p) of the particle electrode $\gamma Al_2O_3xFNCL_{20}$@CuO-400

4. Mechanism of the degradation of organic pollutants from wastewater

Table S5 A summary of the removal efficiency of the metal-Al_2O_3 electrode reported in the preview literature and obtained in this study.

Catalyst	Electrode	Pollutant	Condition	Removal efficient	Reference
γAl_2O_3xFNCL@CuO	A: Pt[e]<br>C: Pt	RhB[a]<br>MB[b]<br>YA[c]	J: 12 V;<br>Catalyst: 150 mg L^{-1};<br>Co: 20 mg L^{-1};<br>RhB pH: 8; MB and YA pH: 5;<br>Time: 40 min;<br>T: 25 ºC	98.82%,<br>99.99%,<br>98.97%.	This study
$CuFe_2O_4/Al_2O_3$	A: Ti/RuO_2<br>C: stain-less-steel	p-nitrophenol	24 mA cm^{-2};<br>CFO/PEs dosage: 0.3 kg L^{-1};<br>Conditions:	80.23%	[3]

			[PNP] = 150 mg L^{-1}; pH: 4.95; T: 30 min		
Bi-Sn-Sb/γ-Al_2O_3	------ ------	tetracycline	I: 0.1 A; Co: 100 mg L^{-1}; Particle dosage:15 g; pH: 5.9; time 180 min	86%	[4]
Co-Ce-Zr/γ-Al_2O_3	A: RuO_2-IrO_2/Ti C: RuO_2-IrO_2/Ti	ciprofloxacin	I: 0.2 A; PEs: 15 g; Co: 100 mg L^{-1}; pH: 6; T: 40 min	52.6%	[5]
Ti-Sn-Sb/γ-Al_2O_3	…. …..	Oxytetracycline total organic carbon	I: 0.5 mA.cm^{-2}; PEs: 20 g; Co: 100 mg L^{-1}; pH: 6; T: 120 min	92.0% 41%	[4]

Noted: [a] Rhodamine B; [b] Methyl blue; [c] yellow acid; [d] Chemical organic demand; [e] Platinum

Table S6 Degradation power of RhB

Name	Number of bonds	Estimated dipole moment	Molecular Formula	Molecular weight m/z	Proposed structure
RhB/P1	36	3.128	$C_{28}H_{32}ClN_2O_3$	443	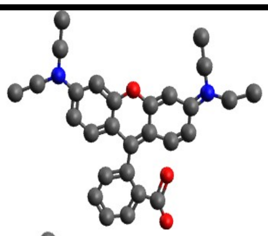
P2	35	5.849	$C_{26}H_{33}N_2O_3$	413	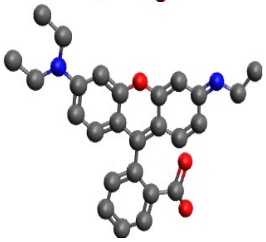

P3	31	4.403	$C_{24}H_{25}NO_3$	297	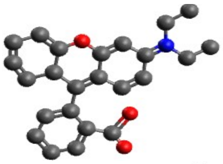
P4	29	5.580	$C_{22}H_{21}NO_3$	327	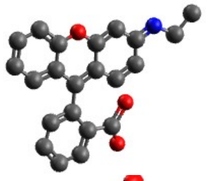
P5	12	4.201	$C_8H_{10}O_4$	167	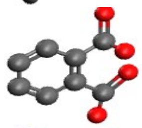
P6	9	1.954	$C_6H_{12}O_3$	132	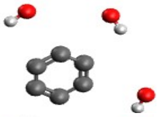
P7	8	2.029	$C_6H_{10}O_2$	114	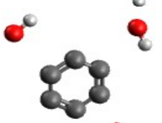
P8			$CO_2 + H_2O$	Mineralization	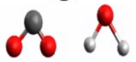

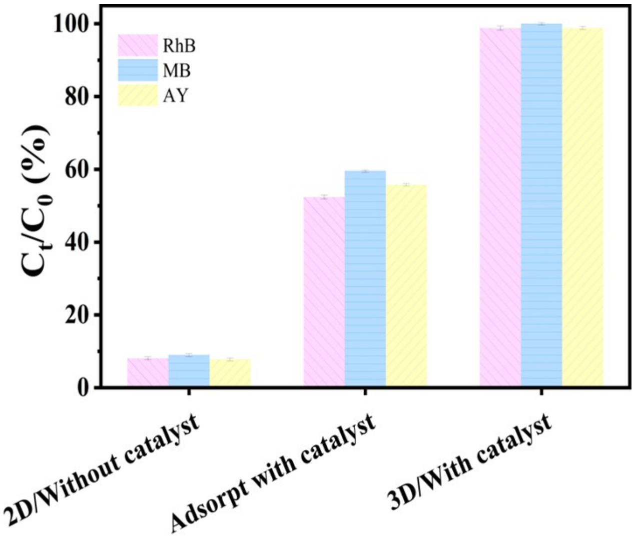

Fig. S10 2D and 3D comparison of the performance in the removal of RhB, MB, and AY with the catalyst $\gamma Al_2O_3xFNL_{20}$@CuO-400, initial condition: 150 mg L^{-1}, $[catalytic]_0$, 20 mg L^{-1} of $[RhB]_0=[MB]_0=[YA]_0$, 12 V, at lab temperature.

Test S6 the difference in system performance

The variation system performance is illustrated in Figure S10, which is based on the comparative results of different systems, adsorption, 2D, and 3D. According to a previous study, the production of ·OH was investigated during the 2D and 3D electrolysis through the RhB degradation. As demonstrated in Section 3.4, Figure 4, a greater number of hydroxyl radicals were produced after 40 minutes in the 3D electrolysis system compared to the 2D system. It has been demonstrated that each particle electrode has the capacity to operate as a micro-electrolytic cell under electrostatic induction, thereby generating an increased number of hydroxyl radicals through water electrolysis in 3D electrolysis[6]. Consequently, the incorporation of suitable particle electrodes within the 3D electrolysis system has been demonstrated to enhance the capacity of indirect

oxidation through the generation of ·OH, thereby augmenting the removal efficiency of pollutants[7].

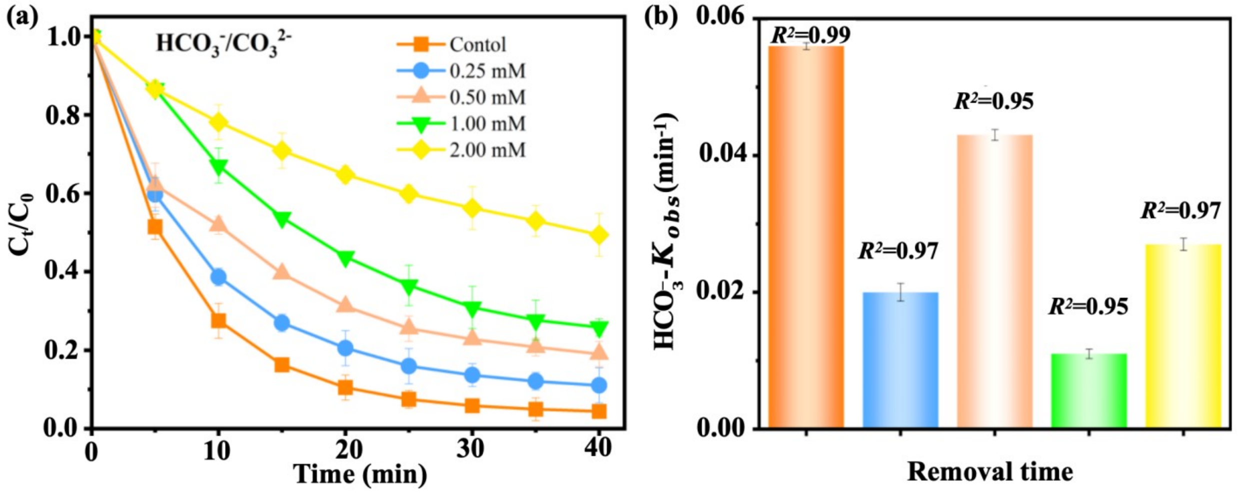

Fig. S11. (a) Effect of HCO_3^{2-} /CO_3^{2-} and the k_{obs}. (b) Durant RhB degradation using the 3D system. Conditions: $[Catalyst]_0 = 150$ mg L^{-1}, $[RhB]_0 = 20$ mg L^{-1}, pH = 8, and 12 V.

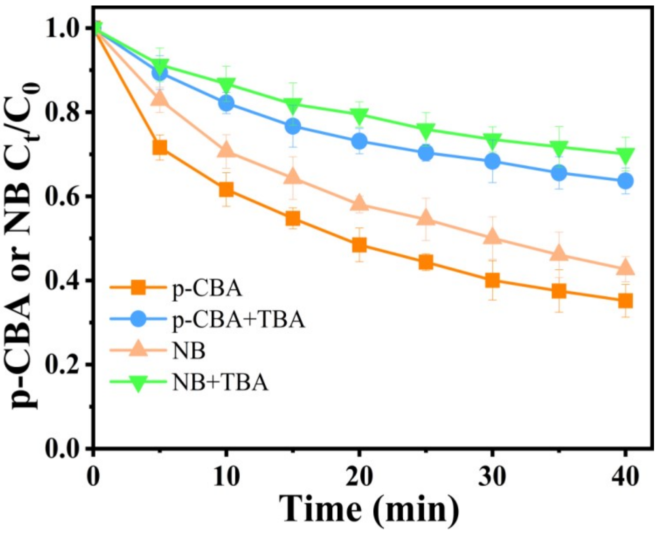

Fig. S12 Oxidation of p-CBA and NB in the 3D electrode reaction without and with the addition of TBA. Conditions: $[Catalyst]_0 = 150$ mg L^{-1}, U= 12V, $[contaminants]_0 = 20$ μM, $[pH]_0 = 7$, T = 25 ºC applied $[TBA]_0 = 100$ mM.

References

1 Uygun HDE, Uygun ZO. Electrochemical Impedance Spectroscopy (EIS) Principles and Biosensing Applications. Handb Nanobioelectrochemistry Appl Devices Biomol Sens. 2021;(2021):919–39.

2 Zuo J, Fu D, Yan P, Wang S, Li Y, Shen L, et al. Synergistic mechanism of surface oxygen vacancies and metal sites on Al-substituted $NiFe_2O_4$ during peroxymonosulfate activation in the solid-water interface for 2,4-D degradation. Chem Eng J. 2024, 480, 147884. //doi.org/10.1016/j.cej.2023.147884

3 Wu X, Song X, Chen H, Yu J. Treatment of phenolic compound wastewater using $CuFe_2O_4/Al_2O_3$ particle electrodes in a three-dimensional electrochemical oxidation system. Environ Technol (United Kingdom). 2021, 42, 393–404. https://doi.org/10.1080/09593330.2020.1760356

4 Sun W, Sun Y, Shah KJ, Chiang PC, Zheng H. Electrocatalytic oxidation of tetracycline by Bi-Sn-Sb/Γ-Al_2O_3 three-dimensional particle electrode. J Hazard Mater. 2019, 370, 24–32. https://doi.org/10.1016/j.jhazmat.2018.09.085

5 Yaxin Liu, Yongwen Ma, Jinquan Wan, Yan Wang, Jian Sun YX. Electrocatalytic oxidation of ciprofloxacin by Co-Ce-Zr/γ-Al_2O_3 three-dimensional particle electrode. Environ Sci Pollut Res. 2021, 28, 43815–43830. https://doi.org/10.1007/s11356-021-13547-9

6 Chao Zhang, Yonghai Jiang, Yunlin Li, Zhongxin Hu, Lei Zhou, Minghua Zhou. Three-dimensional electrochemical process for wastewater treatment: A general review. Chemical

Engineering Journal, 2013, 228, 455-467. http://dx.doi.org/10.1016/j.cej.2013.05.033

7 Pengxiao Liu, Xu Wang, Jing Lu, Ying Li, Bin Hou, Ling Feng. Removal of antipyrine through two-dimensional and three-dimensional electrolysis: comparison, modification, and improvement. Environmental Science and Pollution Research, 2020, 27, 40837-40847. http://dx.doi.org/10.1007/s11356-020-09763-4
